# The ASK trial: a randomised controlled feasibility trial and process evaluation of a complex multicomponent intervention to improve AccesS to living-donor Kidney transplantation

**DOI:** 10.12688/wellcomeopenres.22631.1

**Published:** 2024-10-29

**Authors:** Pippa Bailey, Fergus Caskey, Adarsh Babu, Rachel Ashford, Lindsay Pryce, Lucy Selman, Liise Kayler, Yoav Ben-Shlomo

**Affiliations:** 1 Bristol Medical School: Population Health Sciences, University of Bristol, Bristol, England, UK; 2 Southmead Hospital, North Bristol NHS Trust, Westbury on Trym, England, UK; 3 Gloucestershire Royal Hospital, Gloucester, England, UK; 4 Erie County Medical Center, Buffalo, New York, USA

**Keywords:** Kidney transplantation, Nephrology, Living kidney donation, Feasibility, Randomised controlled trial.

## Abstract

**Background:**

Following identification of barriers to living-donor kidney transplantation, and subsequent development of a multicomponent intervention, we undertook a feasibility trial of the intervention.

**Trial design:**

Two-arm, parallel group, pragmatic, individually-randomised, controlled, feasibility trial, comparing the new intervention with usual care, with a mixed-methods parallel process evaluation. Based at two UK hospitals.

**Participants:**

Individuals were eligible if ≥18 years old, active on the kidney transplant waiting list or had been referred for transplant listing without a contraindication to transplantation. Individuals with a living-donor undergoing surgical assessment were excluded.

**Intervention:**

i) A meeting between a home educator for a dedicated discussion about living-donor kidney transplantation, living kidney donation and potential donors;
ii) A standardized letter from a healthcare professional to a candidate’s potential donors;
iii) A home-based education and family engagement session undertaken by a living kidney donor and a nurse specialist.

**Objective:**

To establish the acceptability and feasibility i) of delivering the developed intervention in existing care pathways, and ii) of undertaking a randomised controlled trial of the intervention.

**Primary outcomes:**

Recruitment and retention.

**Randomisation:**

Participants were randomly allocated 1:1 to i) the intervention or ii) usual care, stratified by site. Minimisation was used to ensure balance in sex, age group, and socioeconomic strata, with probability weighting of 0.8.

**Results:**

183 people were invited to participate. 62 people (34% recruitment) were randomised. 62/62 (100%) completed nurse assessed follow-up at 6 weeks. 51/62 (82%) completed follow-up questionnaires. 3/30 (10%) in the usual care arm and 9/32 (28%) in the intervention arm had individuals ask to be tested for living kidney donation following recruitment to the trial.

**Conclusions:**

Intervention and trial delivery are feasible and acceptable. Findings have informed the design of an effectiveness and cost-effectiveness trial.

**Trial registration:**

ISRCTN Registry ISRCTN10989132 https://doi.org/10.1186/ISRCTN10989132. The trial was registered on 6/11/2020.

## Plain language summary

A living-donor kidney transplant (LDKT) is the best treatment in terms of life-expectancy and quality of life for most people with kidney failure. The UK’s LDKT activity falls behind many other countries. Certain people with kidney disease in the UK are disadvantaged in accessing a LDKT. We want to help people with kidney disease to get a LDKT. In some countries, hospital teams try to help people to find someone who might want to give them a kidney. However, we don’t know if this extra help increases someone’s chance of getting a kidney transplant, or not.

In this study we tested whether a package of extra support was acceptable to patients and their families, and if it could be delivered in the NHS. The extra support included:

1. A meeting between a home educator for a dedicated discussion about living-donor kidney transplantation, living kidney donation and potential donors.
2. A standardized letter from a healthcare professional to a candidate’s potential donors, accompanied by a leaflet about living kidney donation written in simple language without medical jargon.
3. A home-based education and family engagement session undertaken by a living kidney donor and a nurse specialist.

This study was a ‘test’ trial which was designed to find out if a larger trial across the whole of the UK is possible and practical. It was the first step in finding out if this extra support should be funded by the NHS.

34% of those invited to participate agreed to take part. Everyone who took part completed nurse-led follow up and 82% of participants completed follow-up questionnaires. The intervention and trial proved feasible to deliver and acceptable to patients and their families. Findings have informed the design of an effectiveness and cost-effectiveness trial.

## Background

A living-donor kidney transplant (LDKT) is the best treatment in terms of life expectancy and quality of life for people with kidney failure ^1– 3^. In the UK, 86% of adult LDKT recipients are alive 10 years after transplantation, compared to 74% of deceased-donor kidney transplant (DDKT) recipients ^3^. Quality of life is better with a transplant compared to dialysis ^1, 2, 4– 7^. The risks of donating a kidney are very small ^8, 9^. Mortality in living kidney donation is estimated to be between 0.01 and 0.03% ^8, 10, 11^. Perioperative complications, such as wound infection and bleeding, occur in about 7.3% of cases ^10^. Absolute 15-year incidence of kidney failure for most kidney donors is <1%, and the quality of life of donors returns to pre-donation levels after donation ^7, 12– 14^. A LDKT is the most cost-effective treatment for kidney failure ^15, 16^ saving the NHS approximately £20,000 per year compared to dialysis ^17^.

The UK’s LDKT activity falls behind that of many other comparable high-income countries, including The Netherlands, the USA, Israel, South Korea, New Zealand, Switzerland, Canada, Norway, Denmark, and Sweden ^18^. All these countries have active living and deceased donor kidney transplant programmes. In 2019 the number of LDKTs per million population was 15 in the UK, 21 in the USA, and 29 in the Netherlands ^19^. Only 20% of those on the UK national transplant waiting list receive a LDKT each year ^20^ compared to 60% in the Netherlands ^21^. Certain individuals with kidney disease are less likely to receive a LDKT. In the UK, socioeconomic disadvantage is associated with reduced access to living-donor kidney transplantation ^22, 23^: the most socioeconomically deprived people with kidney disease are 60% less likely to receive a LDKT than the least deprived ^22^. There is also ethnic inequity ^22, 23^: people from UK minority ethnic groups constitute 36% of the transplant waiting list but only 18% of LDKT recipients ^3^. Recent evidence suggests ethnic inequity is driven by deprivation ^24^.

Improving equity in living-donor kidney transplantation has been highlighted as a UK and international research priority ^25, 26^. This feasibility trial follows on from a mixed-methods intervention development study ^27^. In this previous work we developed a multicomponent intervention to address previously identified barriers to living-donor kidney transplantation ^27– 32^. The developed intervention addresses transplant candidates’ lack of LDKT knowledge ^28, 29^, lower levels of patient activation ^28, 29^, perceived low levels of social support ^28, 29^, and lower health literacy ^32^. The developed intervention ^27^ is described in the trial protocol ^33^ and below. It comprises the following elements:

1.  A meeting between a home educator and the transplant candidate with a discussion about living-donor kidney transplantation and living kidney donation, and potential donors.

2.  A standardized letter and information sheet from a healthcare professional to a candidate’s potential donors.

3.  A home-based education and family engagement session with the transplant candidates and their family/friends, undertaken by two home educators: a living kidney donor and a nurse specialist.

## Previous trials

A 2017 scoping review identified a lack of evidence-based strategies to increase LDKTs ^34^. The review identified weak evidence that home-based family engagement may be effective ^35^. Writing to potential donors (intervention component 2) is common practice in Norway ^36^ but has never been formally evaluated for effectiveness or harms. Home-based family engagement (intervention component 3) has been evaluated in small RCTs in the Netherlands and the USA ^37, 38^. Both reported more LDKTs in the intervention group compared with control but were underpowered to demonstrate effectiveness of home-visits to increase LDKTs. In the US trial ^38^, 169 participants were recruited. 52% in the home education group received a LDKT, compared to 30% in the control. The sample size was only adequate to detect a 28% difference in the intervention group compared to the control group, at 90% power. In the Dutch RCT ^37^, 163 participants were randomised. 20% of the intervention received a LDKT compared to 5% of the usual care group. The trial was not powered with LDKT receipt as the primary outcome, but with respect to this outcome the sample was adequate to detect a 17% difference in LDKT in the intervention group, at 90% power ^35^. We undertook a random-effects meta-analysis and the pooled estimate shows these small trials do not provide evidence of intervention effectiveness (Risk Ratio for receiving a LDKT 2.08, 95% Confidence Interval 0.69-6.24, p=0.19, unpublished).

Since 2017 nine relevant trials have been registered with WHO trials: 3 pilot RCTs, 5 effectiveness RCTs, 1 non-randomised trial. Six of these studies are based in the USA and three in Canada. None of the trials is based in the UK. The USA already has a higher rate of LDKTs than the UK, and the healthcare system is not comparable to the UK, hence the context of these trials is very different to the UK. None of the interventions under evaluation involve home-based education and engagement, which was highlighted in the 2017 scoping review as the only intervention for which there was weak evidence of effectiveness ^35^. The large Canadian EnAKT LKD ( **En**hance **A**ccess to **K**idney **T**ransplantation and **L**iving **K**idney **D**onation) Cluster RCT (NCT03329521) has just published its findings ^39^. The multicomponent intervention comprised hospital-based patient education and opportunities for transplant recipients and living donors to share experiences with patients, but no family engagement or home-based education: it did not increase access to LDKTs ^39^.

The intervention we have developed combines multiple components which have not been evaluated together as a single intervention that targets multiple barriers to living-donor kidney transplantation. It is not known if delivery of our multicomponent intervention is feasible in the NHS, if the intervention is effective or cost-effective in the UK, or if it reduces inequity in living-donor kidney transplantation which the larger planned RCT aims to determine.

This feasibility trial was designed to gather information on the acceptability, logistics of delivery, and risk of harm of a multicomponent intervention designed to improve equitable access to LDKTs. It aimed to determine if it would be possible to conduct a full-scale effectiveness trial, and to determine the parameters needed to design such a trial. A parallel process evaluation ^34^ investigated the acceptability, implementation and mechanisms of impact of the intervention.

## Objectives

The primary objectives as described in the published protocol ^33^ were:

- to establish the feasibility of delivering the developed intervention in existing NHS care pathways by diverse practitioners at different hospital sites
- to establish if it is possible to undertake an RCT of the intervention.

The secondary objective was to identify ways in which trial processes and intervention delivery could be improved to optimise the future full-scale trial and intervention.

Process evaluation objectives were to assess:

- Acceptability of the intervention to transplant candidates, their family and friends, and healthcare practitioners
- Acceptability of study procedures to participants and healthcare practitioners

Findings have informed the design of a larger pragmatic effectiveness RCT to formally evaluate the effectiveness and cost-effectiveness of the intervention at improving equitable access to living-donor kidney transplantation.

## Methods

### Trial registration

ISRCTN Registry ISRCTN10989132 https://doi.org/10.1186/ISRCTN10989132. The trial was registered on 6/11/2020.

### Trial design

This was a two-arm, parallel group, pragmatic, individually-randomised, controlled, feasibility trial of a multicomponent intervention, comparing the intervention with usual care, with allocation ratio 1:1. A mixed-methods parallel process evaluation was undertaken. The feasibility trial protocol was published in January 2023 ^33^.

## Important changes to methods after feasibility trial commencement

**Figure 1.**
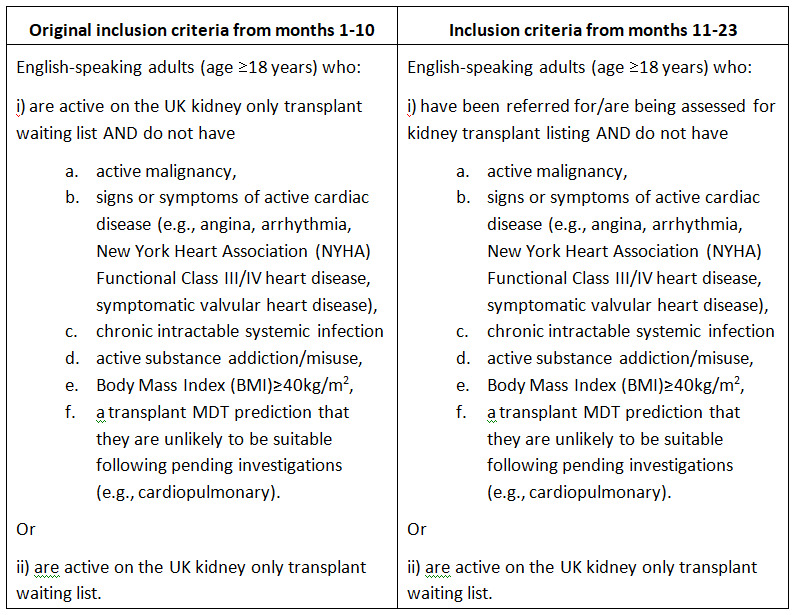
Inclusion criteria.

During the first 10 months of the feasibility trial, recruitment was 26% from the invited population. At the suggestion of the Trial Steering Committee (TSC), we changed our eligibility criteria to include people being assessed for transplantation, not just those on the transplant waiting list. Following this change in month 11 (18/8/22-17/9/22), recruitment improved ( Figure 1).

## Study setting and participants

The feasibility trial took place at two UK NHS hospitals: one hospital is a transplanting centre and one hospital is a non-transplanting referral centre. Both hospitals serve people with all stages of kidney disease. The non-transplanting referral centre does not carry out kidney transplants: patients are referred to two other hospitals for assessment for transplantation and for the transplant surgery. The non-transplanting centre carries out initial assessments of potential living kidney donors but these donors are then assessed by a transplant centre, which also carries out the donor nephrectomy surgery. Both participating hospitals are located in urban areas but serve both urban and rural areas.

***Inclusion and exclusion criteria***

The inclusion criteria are presented in Figure 1. Individuals were excluded from participation if they:

i) had a potential living kidney donor undergoing surgical assessment for donation or approved for kidney donation
ii) lacked the Mental Capacity (as determined by their healthcare team) to consent to participation.

### Ethics and consent

We received Health Research Authority (HRA) approval (IRAS project ID 287507) on 12/2/21 and NHS Research Ethics Committee (REC) approval from West Midlands – South Birmingham Research Ethics Committee (REC reference 21/WM/0003) on 11/2/21. The study adhered to the Declaration of Helsinki ( https://www.wma.net/policies-post/wma-declaration-of-helsinki-ethical-principles-for-medical-research-involving-human-subjects/).

Written consent to trial participation was requested from eligible individuals by research nurses at participating sites. Only patients with the mental capacity to make this decision were able to enter the trial. Consent for process evaluation qualitative interviews was requested at the time of interview. If the interview was undertaken face to face, written consent was requested. If the interview was undertaken over the telephone, consent was audio-recorded, and the researcher who completed the consent form signed to confirm the consent had been recorded. This was approved as part of the study protocol by the REC and HRA.

### Recruitment

Participant identification, invitation, and recruitment have been described in full in the published trial protocol ^33^.

### Randomisation and allocation

Research nurses undertook randomisation of eligible individuals with concealed allocation using internet-based Sealed envelope™ software using minimisation. Participants were allocated 1:1 to i) the intervention arm or ii) the usual care arm, stratified by site to ensure a balance in terms of local differences. Minimisation was used to ensure balance in sex, age group (≤55 years vs >55 years) and socioeconomic strata (English Index of Multiple Deprivation (IMD) ^40^ 2019 deciles 1–5 vs deciles 6–10), with probability weighting of 0.8 in order to reduce predictability. Due to the nature of the intervention, participants and those administering the intervention could not be blinded to allocation.

### Intervention

The intervention has been described in detail in the study protocol ^33^. Two LDKT educators – a nurse specialist and a previous living kidney donor delivered the intervention. It has three components:

**1. **  **Potential donor identification** (adapted from 37): An LDKT educator meets with the participant to discuss LDKT in detail. They create a social network diagram and discussed the initial suitability of family and friends as possible donors. Personal barriers to LDKT are elicited and misinformation addressed.
**2. **  **NHS written outreach to potential donors** (adapted from 36): The LDKT educator and participant agree which family and friends will be sent a:
  • Plain language information sheet about living kidney donation developed with patients and patient charity Kidney Care UK ^27^.
  • Standardized NHS letter introducing the option of living kidney donation with information on how to begin donor assessment.
**3. **  **Home-based family engagement and education** (adapted from 37, 38): A home visit undertaken by a living kidney donor nurse specialist and a trained living kidney donor. The importance of the home in enabling a relaxed environment and avoiding travel costs was stressed during intervention development ^27^ but the meeting can happen as a virtual hybrid to allow people living outside the local area or UK to join. The home visit covers: Healthy kidneys, Kidney disease, Dialysis, Transplantation (DDKT/LDKT), living kidney donation, Personal account of living kidney donation, Psychosocial aspects, Reimbursement.

The living kidney donation nurse specialist and living kidney donor (LKD) were trained in person by the Chief Investigator (CI). This training comprised lectures, video examples and role plays. Online Equality and Diversity training was undertaken at the University of Bristol.

***Intervention resources***

The LDKT educators had a number of resources to share during one-to-one and family engagement sessions. The educators had copies of the standardized ‘Clinical information letter’ to be addressed and sent to a participant’s nominated family and friends, and the adapted Kidney Care UK information leaflet entitled ‘Donating one of your kidneys’. They had iPads with photos of people receiving peritoneal and haemodialysis, photos of transplant and donor nephrectomy scars, and simple pictures illustrating the donation and transplant operations. They had ten educational animation videos presenting an overview of living-donor kidney transplantation (adapted for UK use from animations developed by Dr Liise Kayler Health Resources and Services Administration (HRSA) funded work in the USA ^41^). Animations could be shown during meetings. Links to the online animations were shared at the bottom of the standardized letter, and further links were shared with family and friends via email.

### Intervention modifications

This study was a feasibility trial to finalise the intervention before undertaking a full-scale RCT. The parallel process evaluation collected data on the acceptability of the intervention components alongside delivery. These data were used to make minor changes to the intervention components as part of an iterative development of the resources to optimise acceptability and reach of the intervention prior to evaluation in the full-scale RCT.

### Usual care

In this pragmatic trial, all treatment delivered as part of usual care continued for both trial arms. Usual care typically comprises general kidney replacement therapy education (providing education on dialysis modalities, transplantation) as well as the option of conservative care. This education may be provided in a ‘low clearance/options for kidney care clinic’ or provided in the home environment. Usual care does not include specific education for patients or their families on living-donor kidney transplantation or living kidney donation. Usual care does not involve contacting family and friends with information about kidney donation. No restrictions on concomitant treatments were specified.

### Outcomes

The primary and secondary outcomes and outcome measures were detailed in our protocol ^33^. They are provided again in Table 5 of the results section.

The primary outcome for the later full-scale RCT will be receipt of a LDKT. Secondary outcomes will include the number of potential donors undergoing assessment, the proportion of participants receiving a LDKT, participant patient activation, participant perceived social support, participant transplant/donation knowledge, and cost-effectiveness data.

### Sample size

On average each renal unit in the UK has approximately 75 patients (range 20–250) active on the national kidney transplant waiting list ^42^. The proportion of those listed who do not have a potential living-donor under assessment will be determined in this work. If 80% of those listed do not have a living-donor under review, the eligible population at an ‘average’ site will be approximately 60 individuals. We aimed for a target sample size of 60 patients (30 in each arm). This would allow us to estimate a recruitment rate of 50% with a 95% confidence interval of 41% to 59%. In the 60 randomised patients, we were able to estimate a true rate of follow-up at 3 months of 70% with a 95% confidence interval of 58%–82%.

### Data collection

Trial participants had two standard study contacts and one optional qualitative contact:

1.   Baseline (in person)

2.   Follow-up: questionnaire completion 1–3 days after the home visit or for those not receiving the intervention in the control arm, 4–8 weeks after the baseline assessment.

3.   Qualitative: After the home visit some purposively sampled patient participants will be invited to consider participating in qualitative interviews.

Clinical and laboratory data were extracted from hospital medical records by the research nurses. Patient reported outcome data were collected through questionnaires self-completed by participants. Participant experience of the trial and intervention were collected through qualitative interviews. Compliance with the intervention was recorded by the intervention LDKT educators against a checklist.

***Baseline and follow-up data***

Demographic, social, clinical, and patient reported data were collected by research nurses at the baseline visit (following consent and prior to randomisation). No blood or urine tests were required other than those performed as part of routine care. Details of baseline and follow-up data collection were published in Table 2 and Table 3 of the protocol ^33^.

Data were entered directly onto online electronic case report forms (eCRF), and patient participant questionnaires.

Standardised tools were used:

- Demographics: English Index of Multiple Deprivation (EIMD) 2019 decile ^40^. The EIMD is an area-based measures of deprivation based on routine census data. It comprises seven domains of deprivation (Income, Employment, Health and Disability, Education Skills and Training, Barriers to Housing and Services, Living Environment Deprivation, and Crime) that determine the index score for an area. Areas are then ranked and grouped into deciles: the first quintile represents the greatest deprivation.
- Patient reported mediator and outcome measures: Renal and transplant knowledge (R3K-T) ^43^, social support (ISEL-12) ^44, 45^, Patient Activation (PAM13) ^46, 47^, health literacy (SILS) ^48, 49^, transplant preference, transplant beliefs ^50^, experience talking to family and friends about kidney disease and living donation (binary response questions), quality of life (EQ-5D-5L ^51^). The PAM13 requires a licence granted by Insignia Health®: the Chief Investigator at the University of Bristol holds a license for PAM13 use. As part of the licence agreement, Insignia Health® is provided with non-personally identifiable, individual data captured through use of the PAM Materials, including answers to each of the PAM survey questions, demographic variables, health status and functioning, experience with care/support, self-management behaviours, and health outcomes ("Data") and a royalty-free, worldwide, irrevocable license to use such Data for its product improvement and validation efforts. The trial was registered with EuroQol and fulfilled the conditions for use of the EQ-5D-5L free of charge, in accordance with their terms and conditions (available here: https://euroqol.org/register/obtain-eq-5d/general-conditions-eq-5d-registration/). The SILS questionnaire is freely available for non-commercial use: more information can be found here: https://healthliteracy.bu.edu/sils. The ISEL-12, transplant beliefs and the R3KT questionnaires are freely available to use without licences.

Participants who had been recruited to the trial could choose to withdraw from their allocated treatment. If they withdrew from treatment they could choose to continue follow-up assessments, or withdraw from both treatment and follow-up assessments. Data already collected were kept and clinical outcomes from hospital records were extracted if the participant had consented to this. Participants who withdrew from follow-up assessments had no further contact from the research team regarding the trial.

### Mixed-methods process evaluation

A process evaluation was used to study how the intervention was implemented and to provide information on contextual factors that affect the intervention. It provided information about the uniformity of delivery of the intervention to different participants in different locations, where the “same” intervention may be delivered and received in different ways. The integrated qualitative research provided a more in-depth understanding of how the trial treatments and procedures were delivered and received in practice.

The process evaluation used the following mixed-methods:

- Semi-structured qualitative interviews with participant and non-participant transplant candidates and family members to understand experience and acceptability of intervention, and with practitioners on experiences of delivery.
- Mixed-methods observations of at least two home visits at each study site, assessing fidelity of delivery against a quantitative checklist, and qualitative observation field-notes.
- Quantitative data collection regarding patient participation aggregate data from participating sites on eligible population and invited population, including % of those eligible who were invited to participate and % of those invited who agreed to participation/were recruited.

Fidelity of delivery of the intervention was assessed using implementer self-report against a structured checklist and qualitative interviews with participants and implementers. Fidelity of the home visits was also assessed through structured observations, qualitative observation notes, and participant and implementer qualitative interviews.

Qualitative interviews were used to investigate:

i) **Reasons for non-participation:** individuals who declined to participate in the trial were asked to explain why they didn’t wish to participate, via a telephone interview.
ii) **Experience of the intervention and trial:** Following delivery of the intervention (clinical invitation letters to family and friends, and the home education and engagement session) patient participants, family and friends present at the home visit, and practitioners were invited to an in-depth qualitative interview. These interviews investigated the acceptability of both the intervention and trial methods, and were used to investigate ways of tailoring and optimising the intervention resources for future trials. Participants were purposively sampled aiming for diversity in age, sex, ethnicity, socioeconomic status, and primary renal disease. Participation in the qualitative interviews was optional.

Example interview topic guides were provided as supplemental material with the published trial protocol ^33^.

### Analytical methods

The analysis is described in detail in our published protocol ^33^. Patient-reported outcome scores from questionnaire data were calculated based on the developers’ scoring manuals. Continuous measures are presented as means and standard deviations or medians and ranges depending on their distribution. Categorical data are presented as frequencies and proportions, with 95% confidence intervals. We have described the primary outcome recruitment across socioeconomic position quantiles. Descriptive statistics were used to report the secondary outcomes of interest.

Qualitative interviews were analysed using inductive thematic analysis, as described by Braun and Clarke ^52^ using NVivo software. Data collection and analysis were conducted concurrently, employing an iterative approach. All interviews were coded by the CI (PKB) with a subset dual-coded by RC.

### Data management

Clinical data were stored using REDCap ^53^. Qualitative data were collected as encrypted digital audiofiles then transcribed into word files through secure University of Bristol approved transcription services. Anonymised transcripts were uploaded to NVivo software for analysis and audio files destroyed. Further details of data management are provided in the study protocol ^33^.

### Decision whether to progress to full RCT

A quantitative dashboard with green/amber/red thresholds was proposed to guide decision making regarding progression to a full-scale RCT ( Table 1). Achieving all green targets would almost certainly mean proceeding to the full trial, whereas achieving predominantly red targets would almost certainly indicate that a full-scale RCT is not feasible.

**Table 1.** Progression criteria.

	The recruitment rate per active site month	The % of eligible patients randomised	The % of randomised patients lost to follow-up (no data recorded at hospital site or on national databases) at 3 months
GREEN	3 pts/mth or more	50% or more	30% or less
AMBER	2 pts/mth	25-49%	31-50%
RED	1pts/mth or fewer	Less than 25%	More than 50%

Progression will only occur if the process evaluation interviews find that the intervention and trial processes are acceptable to patients and their families.

This report was written with reference to the CONSORT 2016 guideline extension to randomised pilot and feasibility trials ^49^.

## Results

### Recruitment

Recruitment started in 18/10/21 and ended on 17/9/23 when the target sample size was achieved. Follow-up was for 3 months after randomisation.

**Figure 2.**
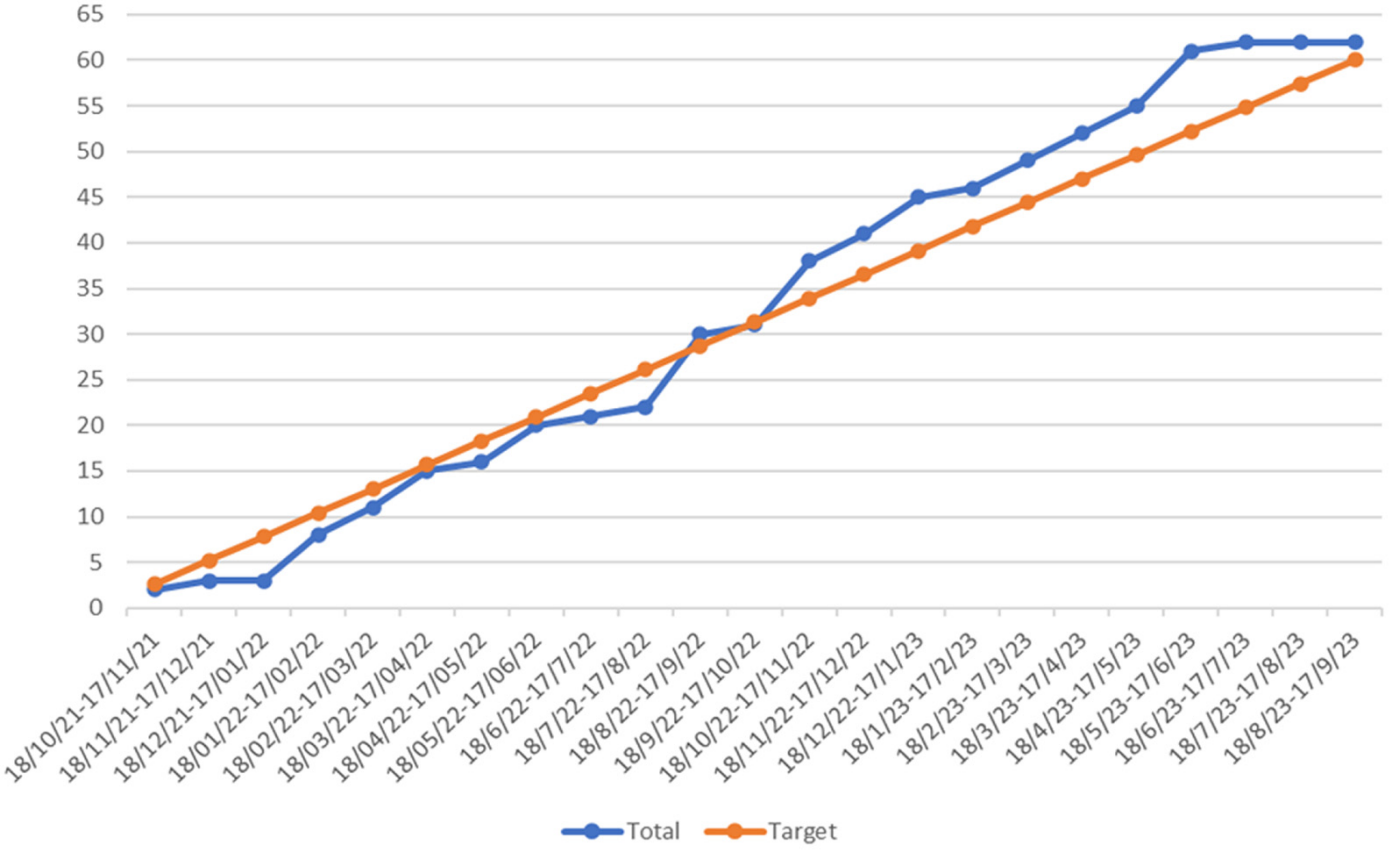
ASK trial recruitment timeline.

During the first 10 months of the feasibility trial, recruitment was 26% from the invited population. At the suggestion of the TSC, we changed our eligibility criteria to include people being assessed for transplantation, not just those on the transplant waiting list. Following this change in month 11 (18/8/22–17/9/22), recruitment improved ( Figure 2).

Participants were representative of the eligible population with respect to sex, age and socioeconomic status ( Table 2). There were no major differences between non-participants and participants. We over-recruited individuals of UK minority ethnicity: 27% of participants versus 22% of the eligible population. This population representative sample, with more than a quarter of participants from UK minority ethnic groups, meant we were able to evaluate the delivery and acceptability of the intervention to people with different ethnic heritage. Reasons for non-participation are detailed in Table 3.

**Table 2.** Equality, diversity, and population representiveness.

Variable	Eligible n=300	Invited n=183	Participants n=62	Non-participants n=121
Sex – number female (%)	95 (32)	56 (31)	18 (29)	38 (31)
Age group (%) ≤25 years 26-45 years 46-65 years <65 years	4 (1) 74 (25) 163 (54) 59 (20)	1 (1) 43 (23) 102 (56) 37 (20)	0 16 (26) 32 (52) 14 (23)	1 (1) 27 (22) 70 (60) 23 (19)
Socioeconomic position EIMD decile ≤ 5 (most deprived) n (%)	154 (51)	97 (53)	30 (48)	67 (55)
Ethnicity Participants from Black, Asian, Other ONS ethnic groups	66 (22)	43 (23)	17 (27)	26 (21)

**Table 3.** Reasons for non-participation.

Reasons for non-participation	Number (%) (n=121)
Reason not disclosed	54 (45)
Could not identify any family/friends to invite/involve	18 (15)
Family/friends already considered/tested and unsuitable/ approached and declined	13 (11)
Doesn’t want to involve family/friends	8 (7)
Doesn’t want a living donor kidney transplant	7 (6)
Doesn’t think intervention would be of benefit	6 (5)
Family abroad, no friends in UK	3 (2)
Already has one or more living kidney donors under review	3 (2)
Family advised against participating	3 (2)
Too unwell to participate in research	2 (2)
Doesn’t want to participate in any research	1 (1)
Competing research – in another study/trial and doesn’t feel time for two	1 (1)
Not happy with study concept	1 (1)
Received deceased donor kidney transplant immediately after invitation	1 (1)

### Baseline data

Trial participant characteristics are presented in Table 4.

**Table 4.** Participant baseline characteristics.

Characteristic	Intervention (n=32)	Usual care (n=30)
**Female sex** (as assigned at birth) (n, %)	10 (31)	8 (27)
**Age group** (n, %) 20–29 years 30–39 years 40–49 years 50–59 years 60–69 years 70–79 years 80–89 years	2 (6) 3 (9) 4 (13) 8 (25) 12(38) 3 (9) 0	1 (3) 1 (3) 12 (40) 6 (20) 7 (23) 3 (10) 0
**Ethnicity** (n, %) White Asian/Asian British Black/Black African/Black Caribbean/Black British Mixed/Multiple ethnic groups Other ethnic group	25 (78) 2 (6) 5 (16) 0 0	20 (67) 3 (10) 7 (23) 0 0
**Religion** (n, %) Christian Muslim Hindu Jewish Sikh Buddhist No religion Other	10 (31) 0 0 0 0 1 (3) 20 (63) 1 (3)	15 (50) 1 (3) 0 0 0 0 12 (40) 2 (7)
**Marital status** (n, %) Single – never married, not living with partner Married/In a civil partnership Living with partner Divorced/Dissolved partnership/Separated Widowed/Bereaved partner	5 (16) 16 (50) 6 (19) 5 (16) 0	6 (20) 15 (50) 5 (17) 3 (10) 1 (3)
**Number of children** (including adopted and step- children) (median, Interquartile range IQR)	2 (2-3)	2 (1.5-3)
**Education level** (n, %) No formal education/training Primary school Secondary school Vocational/Technical/Trade training University undergraduate degree University postgraduate degree	0 0 12 (38) 13 (41) 5 (16) 2 (6)	0 0 8 (27) 11 (37) 8 (27) 3 (10)
**Employment status** (n, %) Full-time employment Part-time employment Temporary employment Unemployed – seeking employment Unemployed – due to sickness/disability Full-time education Home-maker Retired Other	7 (22) 5 (16) 0 1 (3) 9 (28) 0 1 (3) 9 (28) 0	14 (47) 3 (10) 0 0 5 (17) 0 0 8 (27) 0
**IMD decile** (n, %) 1-5 (greater deprivation) 6-10 (less deprivation)	14 (44) 18 (56)	15 (50) 15 (50)
**Housing tenure** (n, %) Own property outright Own property with mortgage Rent privately from a landlord Rent from local authority/housing association Lives somewhere rent free	9 (28) 5 (16) 5 (16) 11 (34) 2 (6)	9 (30) 9 (30) 9 (30) 3 (10) 0
**Car ownership** (n, %) Yes No	21 (66) 11 (34)	26 (87) 4 (13)
**Renal disease stage** (n, %) Pre-emptive (including CKD 4/5T) Haemodialysis – in centre Haemodialysis – at home Peritoneal dialysis	9 (28) 15 (47) 2 (6) 6 (19)	12 (40) 15 (50) 0 3 (10)
**EQ-5Matchability** (n, %) 1-3 Easy 4-6 Moderate 7-10 Difficult Missing	13 (41) 12 (38) 5 (16) 2 (6)	18 (60) 5 (17) 4 (13) 3 (10)
**Calculated reaction frequency** (n, %) 0 >0 Missing	27 (84) 2 (6) 3 (9)	26 (87) 2 (6) 2 (7)
**Number of family members with whom the participant has a ‘close relationship’** (as defined by the participant) (median, IQR)	5 (3-8)	4.5 (2-7)
**Number of friends with whom the participant has a ‘close relationship’** (as defined by the participant) (median, IQR)	4 (2-7)	4 (2-6)

### Outcomes

Primary and secondary outcomes are presented in Table 5. 62/183 (34%, 95% CI 27%-41%) of those eligible and approached consented to randomisation at invitation. 62/62 (100%) completed follow-up CRFs at 4–8 weeks and 3 months. 51/62 (82%, 95% CI 70%-91%) completed follow-up questionnaires at 4–8 weeks.

**Table 5.** Summary of primary and secondary outcomes.

	Outcomes	Findings
**Primary**	• Recruitment • Retention	• Recruitment at 0 weeks: 34% of those eligible and approached who consent to randomisation at invitation. • Retention at 4–8 weeks: 100% complete follow up CRFs at 4–8 weeks and 82% completing follow-up questionnaire at 4–8 weeks (26/32 (81%) in intervention arm, and 25/30 (83%)) in the usual care arm. One participant in the intervention arm disclosed that limited literacy meant they couldn’t read the questions and therefore their responses were deleted) • Retention at 3 months: 100% complete follow up CRFs at 3 months
**Secondary**	• Size of the eligible participant population • Participant and healthcare professional adherence to the intervention/trial • Fidelity of delivery of the intervention • Acceptability of the intervention and trial methods • Barriers to and facilitators of intervention implementation in different settings • Estimates of the effect of the intervention on possible mediators of the intervention • Assessing linkage to UKRR data regarding planned outcome for later main trial (receipt of a LDKT) • Impact on existing healthcare delivery, and potential for the intervention to become a normalised part of everyday care • Cost-drivers.	• Size of the eligible population across 23 months at 2 sites: 300 (248 at transplanting centre, 45 at referral centre). • Adherence to intervention/trial and fidelity of delivery of the intervention assessed at 4–8 weeks: • 97% (n=31) of intervention arm participants received first meeting with LDKT educator • 84% (n=27) of intervention arm participants sent letters to potential donors. Mean 3 letters per intervention arm participant. • 44% (n=14) of intervention arm participants received education and engagement home-visit • 82% of participants completed follow-up questionnaires at 4–8 weeks • 2 protocol deviations reported • Observed fidelity of intervention delivery (see process evaluation) • Acceptability of intervention and trial methods at 10 weeks - assessed through qualitative interviews (see process evaluation) • Barriers and facilitators to recruitment: • Time from first contact with site (submission of request to assess capacity and capability) to green light: Bristol 13 weeks, Gloucester 21 weeks • Time to recruitment of first participant: Bristol 3 weeks, Gloucester 8 weeks • Assessment of screening log: reasons for non-participation in Table 3 and presented in process evaluation findings. • Qualitative interviews with participants, non-participants and healthcare professionals (see Results: Process Evaluation) • Estimates of the effect of the intervention on possible mediators of the intervention at 4–8 weeks in Table 6. • Impact on existing healthcare delivery – activity (LKDs in assessment reported in the ‘Signal of effectiveness’ section below). • Cost-drivers • Mean LDKT educator time per intervention participant: • Intervention delivery: 160 minutes • Travel: 105 minutes • LDKT educators costed at Band 7 NHS Agenda for Change

**Table 6.** Secondary outcome measures.

Variable	Mean change (95% CI) Usual care (n=30)	Mean change (95% CI) Intervention (n=32)	Difference (Intervention-Usual care)	p-value (t-test)
**Perceived social support ^a^** Intervention n=24 Usual care n=21	-0.62 (-2.50, 1.26)	+1.88 (-0.16, 3.91)	+2.49 (-0.23, 5.22)	0.07
**Health literacy ^b^** Intervention n=24 Usual care n=26	0 (-0.28, 0.28)	+0.04 (-0.36, 0.44)	+0.04 (-0.43, 0.51)	0.86
**Knowledge ^c^** Intervention=22 Usual care n=24	+0.58 (-0.25, 1.42)	+1.45 (0.58, 2.33)	+0.87 (-0.30, 2.04)	0.14
**Preference for living donor transplant ^d^** (% difference)	+3.33	0	-3.33	0.82
**Patient activation PAM score ^e^** Intervention n=22 Usual care n=26	+5.07 (0.23, 9.91)	+0.70 (-4.52, 5.92)	-4.37 (-11.30, 2.56)	0.21
**Patient activation PAM level ^e^** Intervention n=22 Usual care n=26	+0.31 (-0.01, 0.63)	+0.18 (-0.28, 0.65)	-0.13 (-0.66, 0.41)	0.64

^a^Perceived social support: positive scores indicate an increase in perceived social support; ^b^Health literacy: positive scores indicate an increase in health literacy; ^c^Knowledge: positive scores indicate an increase in LDKT knowledge; ^d^Preference for LDKT: positive scores indicate an increase in preference for LDKT; ^e^PAM score and level: positive scores indicate an increase in patient activation.

Due to skewness, the perceived social support and health literacy variables were also transformed into normally distributed variables and the analyses repeated. The strength of evidence did not change: Perceived social support squared, difference=+125.52 (-14.61, 265.65), p-value 0.08; Health literacy 1/cubic, difference=-0.13 (-0.37, 0.10), p-value 0.26

### Signal of effectiveness with respect to larger RCT outcome

The feasibility trial was not powered to determine intervention effectiveness, but findings inform the sample-size calculation for the effectiveness RCT. 3/30 (10%) in the usual care arm and 9/32 (28%) in the intervention arm had individuals contact the hospital to be tested for living kidney donation following recruitment to the trial. By the end of the feasibility trial 0/30 in the usual care arm and 2/32 (6%) in the intervention arm had received a LDKT.

### Protocol deviations

There were two protocol deviations: i) a data breach affecting two patients at one site (6/12/21), and ii) a participant’s follow up assessment being undertaken before they had received the intervention (12/10/22).

Both incidents were reported to the sponsor (University of Bristol), the Trial Steering Committee, and the Research and Innovation teams at both participating sites. In addition, the data breach was reported to the REC and HRA who reviewed the action already taken by the site and advised no further action was required.

### Intervention fidelity and adherence

Intervention components

1. **Meeting with a LDKT educator to discuss LDKTs, living kidney donation and potential donors:** 97% (n=31) of intervention arm participants received the initial meeting with a LDKT educator, and sociogram creation.
2. **Written outreach to potential donors:** 84% (n=27) of intervention arm participants sent or gave written information / invitation letters to family or friends.
3. **Home-based family education and engagement:** 47% (n=15) individuals received home-based family education and engagement meeting ( Table 7).
  The reasons intervention arm participants didn’t receive a home-visit are detailed below:
  • 1 participant had people volunteer for donor assessment after the first 2 intervention components had been delivered (home-visit unnecessary).
  • 1 participant felt able to speak to family alone.
  • 8 didn’t want to involve family – relationship difficulties/estranged/couldn’t identify anyone to invite.
  • 2 received a deceased donor transplant before the home visit could be undertaken.
  • 1 chose to have an SPK (simultaneous deceased-donor pancreas and kidney transplant) over a LDKT
  • 3 moved out of area/left the UK
  • 1 uncontactable
  • 1 malignancy diagnosed – no longer eligible for a transplant

**Table 7.** Home-visit content fidelity checklist (intervention delivery team self-report).

Topic	n (%) of 15 intervention participants to whom this was delivered
**What healthy kidneys do**	15 (100)
**Kidney disease – specific to the participant**	12 (80)
**The psychosocial consequences of kidney disease**	13 (87)
**Dialysis (haemodialysis and peritoneal dialysis)**	15 (100)
**Deceased donor kidney transplantation (DDKT)**	15 (100)
**Living donor kidney transplantation (LDKT)**	15 (100)
**The socioeconomic and ethnic differences in access to LDKT**	14 (93)
**Additional advantages and disadvantages of LDKT**	15 (100)
**Living kidney donation**	15 (100)
**The kidney donation operation**	15 (100)
**The psychosocial impact of LDKT for the donor and recipient**	10 (67)
**Lifestyle after kidney donation**	14 (93)
**Common issues after kidney donation**	11 (73
**Financial issues related to kidney donation (reimbursement)**	15 (100)
**How interested individuals can be assessed**	15 (100)

Rather than a formal discussion of the topics ‘The psychosocial consequences of kidney disease’, and ‘Psychosocial impact of LDKT for the donor and recipient’, they were informally covered in the personal account of living kidney donation.

***Animations***

The intervention nurse specialist and living kidney donor had ten animations on a tablet, on living kidney donation and transplantation. The animations were between X and X minutes long and in English. They ranged from 81 seconds to 184 seconds in duration. They could show these to patients at the first meeting, and with families at the home visit, and emailed links to patients, family and friends. The letters to family and friends (intervention component 2) had short URL links to two animations: ‘How to help someone who needs a kidney’ and ‘Basics about kidney donation’. The animations were not well used as a resource. The access metrics for each of the ten animations were:

How to help someone who needs a kidney – 6 views
  Who can be a kidney donor – 4 views
  Basics about kidney donation – 3 views
  What is kidney exchange – 2 views
  Kidney donation day – 1 view
  The kidney donation operation – 1 view
  Lifestyle after kidney donation – 0 views
  Possible complications of kidney donation surgery – 0 views
  How do I become a kidney donor – 0 views
  Healing at home after kidney donation – 0 views

***Harms***

There were 2 deaths (1 in intervention arm and 1 in usual care arm). These were not related to trial participation. One person in the intervention arm was diagnosed with cancer during the trial and was no longer suitable for transplantation. This diagnosis was not related to trial participation.

***Progression to larger RCT***

After review of outcomes against the progression criteria ( Table 1), a decision was made to progress to an effectiveness RCT. 2.7 patients were recruited per month ( green), 21% of those eligible were randomised ( red) but 34% of invited patients were randomised ( amber), and 0% of randomised patients were lost to follow-up at 3 months ( green).

### Process evaluation

21 individuals were interviewed as part of the process evaluation. Qualitative interview participant characteristics are presented in Table 8. Themes and illustrative quotes are provided in Table 9A– Table 9F.

**Table 8.** Qualitative interview participant characteristics.

	n=21
Characteristics	Number
**Interview group**	
** Non-participants**	4
** Patient participants: intervention arm**	4
** Patient participants: usual care arm**	3
** Family/friends: intervention arm**	6
** Healthcare professionals**	4
**Sex**	
** Female**	10
** Male**	11
**Age group (years)**	
** 20–39**	7
** 40–59**	6
** 60–79**	8
**Ethnicity**	
** White**	14
** Other ethnic groups ^1^**	7
**Marital status**	
** Single**	6
** Married/Civil partnership/Long-term partner**	13
** Other (Divorced or widowed/bereaved/missing)**	2
**Patients and family - highest level of education**	n=17
** Secondary school**	2
** Vocational/Technical training**	5
** University undergraduate degree**	4
** University postgraduate degree**	2
** Not disclosed/missing**	4
**Patients and family - employment status**	n=17
** Full or part-time employment (including self-employed)**	8
** Retired and other (e.g. student, homemaker) ^1^**	4
** Other ^1^ (including student, homemaker, unemployed)**	2
** Not disclosed/missing**	3

^1^ Unable to provide information on subgroups due to small numbers in groups risking identification.

**Table 9A.** Non-participant qualitative interviews: themes and illustrative quotes.

Theme	Quotes
Cannot accept perceived donor risk	…there are implications of the live transplant which I haven't come to terms with. Basically, that someone has to donate a kidney! I can't imagine doing anything that would subject those close to me to any kind of risk, however small or however it's rationalised. (Non-participant 4, Male, 60–69 years) I am aware that, the people who donate their kidneys they probably have a harder time getting well because their body has to get used to having one kidney. Whereas mine will just be happily glad to have a decent one. I can remember my mum, she received my sister’s kidney and she was sat there with her lipstick on in hospital looking all jolly and my sister was really feeling poorly. (Non-participant 3, Female, 30–39 years) There’s been about four different people in the family with different sets of kidney issues. So, I was like, even if you’re fit and well now, there is something going on here that you may get something later. … I know tests and stuff are going to be done but…my nan and her brother … they were in retirement age before they were diagnosed with anything to do with kidneys. (Non-participant 2, Female, 30–39 years)
Intervention not perceived to be of benefit	When I have spoken to my friends in the past about living donors they understand the benefits of that but obviously they’re not prepared to put themselves at any risk because of their age and medical condition. I felt that going on the trial and not really having a group of people that I could ask to come and be involved in it would be pointless to a degree. (Non-participant 1, Male, 60–69 years) I mean obviously my sister’s donated one already. My mum was the recipient of that. My nieces, nephews are young. My dad’s had cancer and that just rules him out as well. So, I don’t have a big pool of people ... To be fair my daughter… she said, oh I’ll be a match for you mum but I just tell her that’s she got to save her kidney for her sister. (Non-participant 3, Female, 30–39 years) I think one of the recurring themes we got with people who’d …already had a transplant who were going for a second transplant, and we had a few who were older or maybe a little bit more isolated and they were saying they didn’t feel they had anyone they would want to talk to about a living transplant, so they couldn’t really see the benefit. (Research nurse 1, Female, 20–29 years)
Potential benefit of intervention for non-participants: information gaps identified	Yeah, even if my niece had said no, in the end I’m sure she would have taken part in that just to be there for support and to see whether there was something she could do. Zoom would have been an option definitely. (Non-participant 1, Male, 60–69 years) He’s [partner] always said ‘I’ll donate to you, when can we start the testing?’ It’s really sweet of him but he is ten years older than me. So I don’t even know… I don’t know anything about whether he would be the match or what the criteria is for things like that. (Non-participant 3, Female, 30–39 years) …if I was in that situation [of being a potential donor], I would probably want to clear up what would happen in the event that I donated my organ and then something happened to me. (Non-participant 2, Female, 30–39 years)

**Table 9B.** Trial participant reasons for trial participation: themes and illustrative quotes.

Theme	Quotes
Reason and process of trial understood	When something like that gives you a brief outline of what’s going to happen it’s because everybody’s apprehensive about something like this what’s happening to you … you kind of think oh is it going to be good or is it bad but because you had overall information about what’s going to happen it does put you at ease yeah. (Intervention arm patient 3, Male, 60–69 years) It laid out what options you had regarding the trial whether you wanted to go through with it, and if you did go through it what the steps were that were going to be taken after I’d said yes to that trial…I found that all very useful and very informative and I was able to make a decision based on it. That’s the main thing. (Non-participant 1, Male, 60–69 years) Well, what it does make me think really is that I should have been – maybe I should have been given more information. As I said I wasn’t aware there were two parts to this trial. I thought it was just one... (Usual care arm patient 3, Male, 70–79 years)
Reason for participation	Anything to aid in the research of things really. It was never an option to say no, it’s always good to be a part of a study. (Usual care arm patient 1, Male, 40–49 years) Well, I thought something like that would probably not necessarily help me because I think I were already fixed in but if it helped other people you know for the future you know I’m all for that. (Intervention arm patient 3, Male, 60–69 years) I was hoping to get chosen for the group I ended up in because I think live transplantation is a good thing… I felt being involved in the study I was helping, I hope, I mean I had no idea what the outcome of the study will be. (Intervention arm patient 2, Male, 60–69 years) …you would have thought it would have been UK wide, up and running worldwide. It was just because it sounded like something that was sensible anyway to do. Yes I’ve got kidney problems and I thought I need this. (Intervention arm patient 4, Male, 50–59 years)
Dynamic situation	I: Is there anything [that would have made you take part]? R: I'm guessing that dialysis isn't going to be a very pleasant thing and it's going to be quite intrusive.… I think I'd just hate that. I guess, so far as my wife's concerned, is that it's not just me. The fact of being on dialysis and the restrictions …will actually affect her as well and having a kidney transplant won't only improve my life but it's likely to improve hers as well. (Non-participant 4, Male, 60–69 years) I've said to myself that if my dad were to get worse, I would obviously… I do want to see if I can, if I am…fit enough… (Intervention arm family member 3: daughter of patient, Female, 20–29 years) Basically, I’ve got a little girl on the way…so I didn’t really wanna go turn round to my partner and be, like, ‘Oh, by the way – while you’re recovering from…giving birth, I need to rest because I’m getting me kidney ripped out.’ …I’d rather be there for her …and then, as soon as we’re on our feet and we’re in a routine, then I can do the tests to get things sorted for my dad. (Intervention arm family member 4: son of patient, Male, 20–29 years)

**Table 9C.** Trial participants’ experience of the usual care arm: themes and illustrative quotes.

Theme	Quotes
Experience of allocation to usual care: disappointment, ambivalence, relief	A little bit disappointed, but that soon passed. It was just like, ‘Okay, we'll just crack on and carry on doing what we're doing.’ (Usual care arm patient 1, Male, 40–49 years) No. I'd say I'm not really too fussed myself about which group I go in. (Usual care arm patient 2, Male, 40–49 years) I did find that when you randomised people and they got the, you know, non-intervention, a lot of them were very disappointed … there was a gentleman… and you could tell he just switched off, … he was like ‘well there’s no point in doing this anymore then’. (Research nurse 2, Female, 50–59 years) I think most of the reactions we got were pretty neutral about standard of care, we had one or two who were relieved to be randomised to standard of care because I think they felt it would be less of their time but…a couple were quite disappointed, I think particularly … maybe some of the younger ones… had quite hoped to get intervention. (Research nurse 1, Female, 20–29 years)
Allocation altering behaviour	Personally, these conversations were all in the back of my mind, and it just became more and more to the fore. Regardless, I think it was always going to happen, it was just finding the right words, at the right time, to the right people. … I've got someone going through the process now for a live donation. (Usual care arm patient 1, Male, 40–49 years) …it was just a random social media post, I put it out there to say, ‘Look, I may not be well enough to do the work, and I apologise if you're having to cop the work that I've been allocated. But over the time my health isn't going to get better unless I have a kidney transplant.’ … and people started in-boxing me, I think I had six offers in total, from everyone on my immediate friends list. (Usual care arm patient 1, Male, 40–49 years) I: So, since you’ve agreed to take part in the trial has it made you have any more conversations with anybody about kidney transplantation? R: No, no because I’ve already had a [deceased donor] transplant. (Usual care arm patient 3, Male, 70–79 years) I: Did the trial have any impact on having any conversations with family about kidney donations? R: Not really, in terms of family, so me and my dad and my brother. My brother lives in [Place named redacted] so I don't really see him very often. (Usual care arm patient 2, Male, 40–49 years)

**Table 9D.** Trial participants’ experience of the intervention arm: themes and illustrative quotes.

Theme	Quotes
Overcoming a culture of silence	It has helped a lot because I don’t open up much to the children … they’re all men …in their 30’s … but it helped them understand what I’m going through … you know coming from somebody outside our family group … because I look fit and healthy, and it really drove it home to them that I’m not… and that helps a lot because then they can actually change their behaviour, show empathy towards your situation. (Intervention arm patient 3, Male, 60–69 years) I didn't know my biggest sister, one of my nephews, … he got kidney problems but he didn’t tell nobody. So when I tell my sister and she did come to me and say to me, yes, your nephew’s got it. But nobody say anything. Kept it secret until the other day … he came out and talked to me and I talked to his parents about it. (Intervention arm patient 4, Male, 50–59 years) It hasn't helped me find a kidney but I think it's helped in just talking about it. As I said, I've had more conversations and I do talk more about the dialysis as well. So I think I've benefited in it's given me a chance to talk, it’s given me an excuse to talk about more aspects of it. (Intervention arm patient 1, Male, 50–59 years)
Addressing misinformation	I’ve been told a woman or man could give me a kidney. (Intervention arm patient 4, Male, 50–59 years) They inform you about different things because I were under the impression that because I’m black I thought that it would have to be a black donor but apparently that’s not … what they said to me was totally… blew what I thought out of the water basically (Intervention arm patient 3, Male, 60–69 years) One thing my dad didn’t know – which was a bit sad to hear – is … dialysis was not ‘a fix,’ … he didn’t realise that, with dialysis… things can get worse, still. When they said it, I seen his face dropped, and then I said to him after they left, ‘What’s wrong?’ and he was like, ‘Oh, I thought, when you’re on dialysis, it just keeps it stable.’ (Intervention arm family member 4: son of patient, Male, 20–29 years) I feel like some people were probably more primed for dialysis. That was the, kind of, next natural step. Some people did say things like, ‘Well, my kidney number is 17, so when it gets down, then they’ll start dialysis, then I can think about [transplants].’ And sort of exploring this that, ‘Actually, you could bypass dialysis…you could be lining this up, and bypass that altogether.’ (Intervention delivery nurse, Female, 30–39 years)
Protection from being in a study	So yeah I did feel a bit uncomfortable about giving them to them because it’s not the research fault it’s me as a person it’s me I just don’t like asking for help with anything... (Intervention arm patient 3, Male, 60–69 years) I said to people, if you don't like what it [the letter] is saying tell me, because this is a study. It’s not a - I'm not asking you for a kidney. I'm asking you to do this study, which is slightly disingenuous, yes. I do think it let me get away with it if you like being in the study. (Intervention arm patient 1, Male, 50–59 years) …the first person I saw, they were quite quick to say that they were going to use the trial as a way in to get their family together. They wanted to hide behind the trial, which I found quite helpful, because then I could always put that… give people options of how to suggest it to family, that they could either ask for it themselves, or… I said, ‘You’re very welcome to blame it on us.’ (Intervention delivery nurse, Female, 30–39 years)
Importance of the home	It makes you feel more at ease about it. I think it if was in the hospital I think it would have been a bit more iffy, a bit more clinical, but being at home in your own environment it makes it a lot more at ease and you can probably open up a lot more than if you were in the hospital. … hospital for me is an alien environment and I don’t like hospital at best of times. (Intervention arm patient 3, Male, 60–69 years) …personally I felt it was better being in the home than actually go in to a side room of a hospital, an office having a chat, we were here on our own terms. (Intervention arm patient 2, Male, 60–69 years) I think people were probably more comfortable and relaxed to be in their own homes. … I think it meant that when we finished, I think it seemed like there would be conversations that would then carry on. It wasn’t, ‘Right. I’ve got to lock this door now, because it’s a meeting room, and you’ve all got to go.’ I think things probably organically might have carried on, and then come to a natural end. (Intervention delivery nurse, Female, 30–39 years)

**Table 9E.** Family and friends’ experience of the intervention: themes and illustrative quotes.

Theme	Quotes
Family tensions	… when we had the zoom call with the children I didn’t get involved I stayed upstairs because I think I would have got angry with the kids for not putting themselves forward because for me it’s a no brainer you know if it were any of my family or any of my close friends it just is a no brainer it’s something that I would do and not even think about it…(Intervention arm family member 6: wife of patient, Female, 60–69 years) I think my brother should have been there but that’s on him. He buries his head in the sand and he doesn’t want to know about it. (Intervention arm family member 1: daughter of patient, Female, 20–29 years) I did feel alone in the situation because I felt I didn’t really have anyone to speak to about it and then when I'm speaking to my mum about it, my mum is against it all because of the bias of my mum and my dad not being together (Intervention arm family member 3: daughter of patient, Female, 20–29 years)
Importance of both lived and professional experience	It was really good to hear somebody on there that had given a kidney. I found that was really helpful and then obviously you’ve got the nurse there which she knows the facts and everything like that. (Intervention arm family member 1: daughter of patient, Female, 20–29 years) I think the combination of the two was brilliant…because you’ve got the nurse side of it – of ‘How does the operation work – what are the risks – what’s this, what’s that?’ – …but, from a personal point of view, [Home visit nurse] didn’t know what it’s like to do it….so, between the two of them, they’re a brilliant team. (Intervention arm family member 4: son of patient, Male, 20–29 years) It was also really nice to have the [living kidney donor] there to discuss her experience of it all. That was nice because she was very honest as well, she was saying at the time I didn’t want to do it and all the trouble that she faced with her employment and everything. That was a nice insight to hear and get a real holistic viewpoint of things. (Intervention arm family member 1: daughter of patient, Female, 20–29 years)
Patient gatekeepers	He’s always said, no, I don’t want one of your kidneys but in the chat the other day he actually spoke about it and at the start he felt like a burden to ask somebody for their kidney because obviously it’s a big ask, isn’t it? And he said, now with the research and things like that he’s realised it’s not a burden and he doesn’t feel like he’s begging somebody for a kidney. (Intervention arm family member 1: daughter of patient, Female, 20–29 years) I think it forced us to go through this scenario of – look me and [Name redacted] are here for you; we’re happy to do this. You need to stop worrying about us too much and let us help… I think from that point onwards there was a little bit of step change in his attitude towards it. (Intervention arm family member 1: daughter of patient, Female, 20–29 years) He told me that, ‘No, you’re too young to be a donor’ – this, and stuff like that. When you guys come round and said, ‘No, that’s not the case,’ it was a bit of a, ‘Hmm, are you lying, or did you genuinely not know?’ (Intervention arm family member 5: son of patient, Male, 30–39 years)

**Table 9F.** Intervention optimisation: themes and illustrative quotes.

Theme	Quotes
Tailored donor	I don’t really think there’s anything you could have done better, but, maybe, if you had the opportunity to bring someone [a donor] of my own age … but, obviously, that’s not always possible, is it? (Intervention arm family member 4: son of patient, Male, 20–29 years) I feel because my dad’s generation and what they’re used to, for that maybe having someone of Jamaican heritage… I feel like if it was somebody of the same ethnicity maybe it would have been more like… how can I explain it? I guess because we have the same kind of culture, and the way that our family structures are quite similar…would have probably been a better viewpoint and like okay well this lady or this gentleman have gone through the same things that I have gone through (Intervention arm family member: daughter of patient, Female, 20–29 years) I do a lot of running, sort of sports events and stuff like that and I wanted to know how much it would affect that and that sort of stuff and I don’t think that that information was necessarily readily available then. (Intervention arm family member 5: son of patient, Male, 30–39 years)
Norway letter	Yeah, a little bit more personalised letter and that would make all the difference. I think we all receive generic letters from companies and …I think in something like this, you just don’t want to feel like a number. (Intervention arm family member 5: son of patient, Male, 30–39 years) I know one of my more recent ones, she wrote a cover letter with it, her own cover letter. I would, sort of, always lead with the fact that the letter was optional, like it all was, and that for some people, they really liked the letter, because again, it wasn’t from them, it was from an official body. Other people found it really too direct. (Intervention delivery nurse, Female, 30–39 years) I sensed some patients felt the letter might need the case for a transplant stated less strongly, although only [Patient name redacted] said this overtly. I’d make the focus more about getting information. (Intervention living donor, Female, 60–69 years)
KCUK information leaflet – useful but not sufficient	I think that leaflet being called ‘Donating One of Your Kidneys’ then slightly [challenged] what we were saying, that it wasn’t about donating. So, perhaps that could be retitled ‘Information About Kidney Donation’. Rather than to give it, ‘…the finger is pointing at me.’ (Intervention delivery nurse, Female, 30–39 years) I’ll be honest…the leaflets are good once you’ve spoke to people like [Home visit living kidney donor and nurse]…because, then, if you think, ‘Oh, what was it they said about this?’…you’ve got something to refer back to… If you just give me the leaflet …I’ll be honest, I probably wouldn’t have read it. (Intervention arm family member 4: son of patient, Male, 20–29 years) I think they was useful but some of the words I couldn’t actually read, I was like what do they mean, you know. You’ve got to be a doctor to understand it. I got my little magic pen and it reads the words for you and it tells you what it means. (Intervention arm patient 4, Male, 50–59 years)
Animations poorly accessed	I remember [Home visit living kidney donor] having a tablet [ipad] and kind of went through some photos and erm, yeah, but I don’t remember any animations as such. (Intervention arm family member 5: son of patient, Male, 30–39 years) We hardly used the animations. We mentioned them if people wanted them but people didn't ask for them. Probably because the face-to-face thing is what really engaged people and it's just another thing to look at, and they were quite simple so … they possibly didn't answer the sort of questions that people want to ask, and then if you've got someone there to ask… (Intervention living donor, Female, 60–69 years) To get the same amount of information in the animations that you got in the leaflet, you’d have to watch about seven or eight, and they’re each 2 or 3 minutes. I would show some at the beginning of the session, but I don’t know if it was my personal discomfort with sitting there like a lemon watching it. I would … say, ‘Shall I email this to you, and you can watch it at your leisure?’ So, I always emailed … the first two, and actually, no one came back to me to ask for more…maybe between with the session and the leaflet, there was nothing else to add. (Intervention delivery nurse, Female, 30–39 years)

***Non-participants***

Non-participants reported deciding against participation in the trial for two main reasons: i) they could not accept the perceived donor risks, and ii) the intervention was not perceived to be of benefit. Those not willing to accept family and friends donating could not accept the perceived risks to donors, despite these being small:

*“I can't imagine doing anything that would subject those close to me to be at any kind of risk, however small or however it's rationalised. (Non-participant 4, Male, 60–69 years)*

Those who perceived the intervention to be of no benefit reported that they had already considered living donation and reported a mixture of reasons why their family and friends were unsuitable. Some participants had discussed living donation with friends who had then not offered to donate, whilst others had considered family and friends but ruled them out without approaching them. One non-participant had a familial renal disease and therefore family members had kidney disease, had already donated a kidney, or were being ‘reserved’ for another intended recipient in the family:

*“To be fair my daughter… she said, oh I’ll be a match for you mum but I just tell her that’s she got to save her kidney for her sister.” (Non-participant 3, Female, 30–39 years)*

Some non-participants suggested that the intervention was akin to asking people to donate, and ‘they just couldn’t ask’. For one person their explanation of family and friends being unsuitable appeared to be secondary to the fact that they didn’t want to ask anybody to consider donation:

*“So, I don’t have a big pool of people and also I would never ask people. I would just not ask…” (Non-participant 3, Female, 30–39 years)*

People who were waiting for a second or third transplant had often been through the process of looking for a living donor for their first transplant and therefore didn’t see a potential benefit from the intervention. However, there was evidence that some of those who perceived no benefit from the intervention had gaps in their understanding that, had they been addressed, could have facilitated access to a LDKT. One individual reported that their partner had offered to donate:

*“but he is ten years older than me” and she didn’t “know anything about whether he would be a match or what the criteria is for things like that” (Non-participant 3, Female, 30–39 years).*

This suggested that a patient’s ‘screening’ of family and friends as potential donors is limited by their understanding regarding suitability, which the intervention in this trial addresses. When considering changes to recruitment in the effectiveness trial, focusing on the potential information gain, and checking that people know all they need to know about LDKT, may be of benefit.

***Trial participation***

Most interviewees appeared to have understood the information sheet and the aim of the study, reporting that it provided *“overall information about what’s going to happen”* (Intervention arm patient 3, Male, 60–69 years). There was an exception to this: one participant reported not being aware that there were two parts to the trial, and after understanding this stated *“maybe I should have been given more information”* (Usual care arm patient 3, Male, 70–79 years)

Some people participated because they were hoping to get the intervention, and felt this would benefit them. Some individuals viewed participation as benevolent: viewing research as an opportunity to ‘help’ other people. Others felt they had *“nothing to lose”* (Usual care arm patient 3, Male, 70–79 years).

Participants and non-participants recognised that the situation was dynamic, and that both the decision to participate and the impact of the intervention may change over time. One individual who was not yet receiving dialysis treatment suggested that starting haemodialysis might change his willingness to consider a LDKT. This was echoed by a family member who attended a home visit, who said that they would consider donation *“if my dad were to get worse”* (Intervention arm family member 3: daughter of patient, Female, 20–29 years).

Similarly, family members reported that their reasons for not wanting to donate a kidney at present might change and with that their decision about donation might change. The investment currently wasn’t perceived as ‘wasted’ if a family member hadn’t donated ‘for this transplant’. After a participant in the intervention arm received a DDKT, his wife described how his children were still considering donating for a future transplant:

*“I have to say they’re still saying, if this kidney fails or if he needs a kidney in the future, they would still consider it.”( Intervention arm family member 2: wife of patient, Female, 60–69 years)*

***Experience of the usual care arm***

The response to allocation to usual care was mixed. Some participants reported being disappointed, something witnessed by the research nurses who undertook randomisation. However, the research nurses at the two sites both reported that some individuals appeared relieved to receive usual care. One of the research nurses described the usual care arm as ‘non-intervention’ but suggested that to avoid people feeling ‘abandoned’, usual care should be emphasised as a continuation of current standard care:

*“if you don’t get the intervention, … you’ll still be supported by people who are working you up on transplant list, so you’re not just on your own”* (Research nurse 2, Female, 50–59 years).

There was no evidence of contamination, that is, there was no evidence that usual care arm participants accessed the resources, healthcare professionals or home visit of the intervention. However, one usual care participant reported a behaviour change as a result of allocation to usual care. He reported that following disappointment about his trial allocation conversations about transplantation and donation which had been in the back of his mind came *“more and more to the fore”* and as a result he spoke to people about his kidney disease and put out a social media post, resulting in six people offering to be tested for kidney donation. Although this was the only example, as already highlighted a greater proportion of individuals in the usual care arm received DDKTs than the intervention arm, which may have limited opportunities for usual care arm participants to pursue a LDKT.

***Experience of the intervention arm: patients***

Patients reported the intervention helped to overcome cultures of silence within families and with their wider social network including work colleagues. Patients described the home engagement visit helping their family and friends gain a better understanding of their kidney disease, treatments and the impact on their life and work. One patient described finding out that another close family member also had advanced kidney disease, but that neither had known that the other had kidney disease. Patients described a benefit from this greater understanding and increased empathy, even if this didn’t facilitate a living donor transplant.

The process evaluation suggested the intervention might be effective at increasing access to LDKTs: family and friends were reported as offering to donate after they had received the intervention. This included a case in which a father who had previously refused his son’s offer to donate, changed his mind, and the son donated. The recipient described to a research nurse going from ‘80% against’ to ‘80% for’ a LDKT from his son.

The intervention addressed misinformation and misunderstandings about LDKTs. For example, patients described learning that a donor of any sex and ethnicity could be suitable for them. Relatives described learning that someone’s health generally deteriorates on dialysis. The intervention nurse specialist described patients appearing to have an understanding that dialysis needed to precede transplantation, and that it was new information to learn that a LDKT could help them to ‘bypass dialysis’ altogether.

Some participants reported feeling uncomfortable about giving the letter to family and friends, but recognised that it was difficult because the conversation about transplantation and donation was a difficult one, and it was not something that could necessarily be avoided. A participant reported using the research trial as something to ‘hide behind’: he was able to say that he was asking people to take part in a research study not to donate a kidney, which he said was helpful but felt disingenuous. The intervention nurse specialist reported other participants doing the same.

The importance of the home setting for the family engagement visit was emphasised: it helped patients and their families to feel relaxed and comfortable, away from the clinical environment.

No patient qualitative interview participants reported any harms from the intervention. One research nurse reported that two participants had reported disappointment to her that the intervention hadn’t resulted in family and friends offering to donate a kidney:

*“I think it left him feeling quite low because he’d kind of re-approached them [family] and you know, they had said that they didn’t want to do that. (Research nurse 1, Female, 20–29 years)”*

***Experience of the intervention: family and friends***

The home visit itself was viewed positively by all interviewees, with participants describing it as informal, relaxed, and ‘a laugh’. The family of patients who had received a home therapy reported being *“used to people [healthcare professionals] coming [into the home]”* (Intervention arm family member 2: wife of patient, Female, 60–69 years). The benefits of group dynamics were noted: one family member described a friend of her Dad’s being present at the meeting, asking a question she wanted to ask but felt uncomfortable doing so.

Tensions and difficult relationships within families were apparent and described by family participants, which the home visit highlighted:

*“I think my brother should have been there but that’s on him. He buries his head in the sand and he doesn’t want to know about it.” (Intervention arm family member 1: daughter of patient, Female, 20–29 years)*

Family and friends had self-identified knowledge deficits regarding donation and transplantation, and the home visit addressed these gaps. Family members described gaining a ‘holistic’ understanding of donation with ‘medical facts’ from the nurse specialist and ‘lived experience’ from the donor. Patients were identified as gatekeepers to the whole process: family members described their relative with kidney disease as having prevented or avoided discussions about donation, which the home visit enabled, with family members reporting witnessing a change in their relatives’ attitude to accepting a LDKT.

Family members were explicitly asked if they felt pressured to donate. One family member described faith and trust in the assessment process that individuals who may not be happy to donate would not be allowed to donate. One family member explained that they hadn’t realised they could start assessment for donation without informing their intended recipient: learning this reduced the pressure on them. Two individuals, who were both under the age of 30 and children of their intended recipients, reported being put in a difficult situation, feeling that if they did or didn’t donate there would be potential negative consequences. Reassuringly, one daughter of a patient explained that after feeling like she had to respond to the ‘Norway letter’, she phoned the living donor coordinators who provided reassurance that she didn’t have to donate. This was prior to the home visit, at which she then gained further information about donation, and realised that it was not just her who had been given information on donation, which took some of the pressure off her. She further explained that it wasn’t a bad thing for her to feel like she needed to make a decision about donation, but admitted that this was a difficult decision.

### Intervention optimisation

Family attendees at **the home visit** found meeting a living donor extremely helpful but suggested that the more similar the donor was to them, with respect to age, culture, and lifestyle, the better. One participant recognised that ‘that’s not always possible’ and the intervention nurse noted that *“meeting any donor was better than not meeting a donor at all”* (Intervention delivery nurse, Female, 30–39 years). We specifically explored whether the intervention needed to be delivered by people who shared the cultural and ethnic heritage of the patient and/or their family. One family member felt that older generations may have preferred hearing from someone who shared their cultural and ethnic experiences, but that this wasn’t essential for her.

**The Norway letter** was recognised as raising a difficult topic for people who were not aware about or had not considered donation. One patient wrote her own personal cover letters to accompany the formal letter. One family member felt the letter should be a little bit more personalised and a little less generic. One patient told the intervention delivery team that the need for a transplant should be ‘stated less strongly’.

Overall, **the leaflet** was considered useful but not sufficient. A family member described how it was useful to have something to refer back to after meeting with the intervention team, but had he been given this without a meeting, he wouldn’t have read it. A patient and the intervention nurse suggested that the leaflet implied people were already considering kidney donation, and that for simple information giving it should be retitled as ‘Information about kidney donation’. The leaflet was written in plain language but this was not suitable for some participants with limited literacy who needed assistance reading it. As reported earlier **the animations** were very rarely accessed by participants. Participants were provided with links to the animations on a letter and via email. The intervention team described difficulty using them within the home visit setting, and speculated that this was due to them being superfluous when people had all the required information delivered to them in a personalised way at the home visit, with the leaflet for reference.

## Discussion

This feasibility trial demonstrated that both the multicomponent intervention and trial processes are i) acceptable to patients, their families and healthcare professionals, and ii) deliverable in transplanting and non-transplanting referral NHS hospitals in the UK. The study demonstrated that a larger effectiveness trial is feasible, and after review of outcomes against the progression criteria ( Table 1), a decision was made to progress to an effectiveness RCT.

Assessment of secondary outcomes and potential intervention effect-mediators provided a signal regarding the mechanism of impact of the intervention. There was no evidence from this study that the intervention increased a participant’s health literacy or patient activation: the intervention is not expected to change a participant’s health literacy or patient activation, but rather to provide work around solutions to these barriers to living-donor kidney transplantation. The intervention does aim to increase social support and knowledge, and this underpowered feasibility study provided weak evidence that it does this.

### Sample size for effectiveness trial

3/30 (10%) in the usual care arm had individuals contact the hospital to be tested for living kidney donation following recruitment to the trial. If 10% of people in the usual care arm receive a LDKT, to detect a +10% difference in the intervention arm with α=0.05 and power=0.9 we will require 592 participants, allowing for 10% attrition. The 10% difference is a conservative estimate of the anticipated difference between the proportion of patients receiving a LDKT in the intervention arm compared to the usual care arm. It is smaller than the difference observed in the (underpowered) Dutch and US RCTs of a home education intervention. In the Dutch RCT there was a 15% difference between the proportion of LDKTs in the intervention versus control arm ^4^. The US RCT reported a 22% difference ^5^. In our feasibility trial, the short-term follow up means the proportion of LDKTs received was not measured, but 28% of participants in the intervention arm had a person under assessment for kidney donation (in donor assessment) versus 10% in the usual care arm i.e. an 18% difference. Given the feasibility trial recruitment of 18 participants/centre/year, 20 renal units recruiting over 20 active site months will provide the required sample size.

### Future work

Funding has been secured for the future definitive trial (NIHR HSDR Reference: NIHR160325) which is expected to start in 2025. As a result of the feasibility trial, the following changes have been made to the protocol:

- Eligibility criteria changed as detailed in Figure 1.
- Animations dropped as intervention resource.
- Questionnaire shortened.
- To allow a degree of tailoring of the LKD undertaking the home visit to the participant and family’s particular needs, rather than a single LKDs being employed at each site, we will identify and train a pool of LKDs (up to 5) at each site with home visit selection informed by characteristics important to the patient and family when requested and possible.
- Given a patient and family’s situation being dynamic, as highlighted in the process evaluation, we will consider repeat invitation to participation in the trial, with re-invitation happening no sooner than 6 months after the initial invitation.
- Again, because a patient and family’s situation is dynamic, the impact of the intervention on the likelihood of a family member donating and a person receiving a LDKT, may not be realised for many years, even for a subsequent transplant. Primary outcomes will be recorded 18 months after randomisation in the definitive trial, and we will consent for data linkage to allow extraction from existing transplant registries beyond 18 months.

### Strengths and limitations

Our feasibility trial met the aims and objectives set out in our protocol. We achieved our target population ahead of schedule. We achieved a population representative sample and an over-representation of participants from UK ethnic minority groups. However, the feasibility trial has some limitations. It was undertaken at two hospital sites. Although the sites included a transplanting hospital and a referral hospital, we cannot at this stage be certain that all barriers to and facilitators of intervention and trial delivery have been identified. Therefore, a process evaluation is essential alongside the planned effectiveness RCT. Whilst a diverse sample was achieved within the eligible population at the participating sites, there is obviously still wider diversity nationally. The planned effectiveness trial will be undertaken in conjunction with the UK’s Centre for Ethnic Health Research (CEHR) to ensure that intervention delivery nurses receive training in cultural competency, and to ensure that the delivery is culturally tailored when required.

### Protocol

The feasibility trial protocol is published as an open-access publication: Bailey PK, Caskey FJ, MacNeill S, Ashford R, Pryce L, Kayler L, Ben-Shlomo Y. Investigating strategies to improve AccesS to Kidney transplantation (the ASK trial): a protocol for a feasibility randomised controlled trial with parallel process evaluation. Pilot Feasibility Stud. 2023;9(1):13. doi: 10.1186/s40814-023-01241-1. https://pilotfeasibilitystudies.biomedcentral.com/articles/10.1186/s40814-023-01241-1

## Data Availability

Study documents are available at the University of Bristol Data Repository: https://data.bris.ac.uk/data ASK feasibility trial documents ( https://doi.org/10.5523/bris.1u5ooi0iqmb5c26zwim8l7e8rm). This repository contains: Consent forms Trial information sheet Trial questionnaire Qualitative interview demographics forms Qualitative interview information sheets Qualitative interview topic guides These documents are published as ‘open access’ ^54^. The ASK feasibility trial: Wellcome Open Research CONSORT checklist ( https://doi.org/10.5523/bris.1m3uhbdfdrykh27iij5xck41le) ^55^. This repository contains: Wellcome Open Research CONSORT checklist. This document is published as ‘open access’. ASK feasibility trial: CONSORT documents ( https://doi.org/10.5523/bris.2iq6jzfkl6e1x2j1qgfbd2kkbb) ^56^ This repository contains: Feasibility trial protocol CONSORT flow diagram CONSORT checklist (page numbers as per submitted manuscript) These documents are published as ‘open access’. Quantitative data are available at the University of Bristol Data Repository: https://data.bris.ac.uk/data ASK feasibility trial: QUANT ( https://doi.org/10.5523/bris.2b9vlo0wejnsh2nfoa6fka66cx) ^57^. Two participants did not consent to data sharing, so their data have not been uploaded. This project contains the following underlying data: The ASK feasibility trial dataset (excel) The ASK trial questionnaire (word) The trial consent form and information sheet stated requested permission to share anonymised data with other researchers. In order to fulfil this condition, the dataset must be published as ‘restricted access’. Researchers can request permission to access via the data repository. Quantitative data are available at the University of Bristol Data Repository: https://data.bris.ac.uk/data ASK feasibility trial: QUAL ( https://doi.org/10.5523/bris.1qm9yblprxuj2qh3o0a2yylgg) ^58^. Anonymised transcripts of the interviews have been uploaded to the University of Bristol’s Research Data Repository. Two participants did not consent to data sharing, so their interview transcripts have not been uploaded. Although the qualitative transcripts have been anonymised, as personal and sensitive issues have been discussed we cannot rule out the risk of identification, and therefore access to these transcripts is controlled. Individual researchers will need to request access to the controlled data through the University of Bristol via the Data Access Committee (DAC) for approval, before data can be shared after their host institution has signed a Data Access Agreement. The procedure for accessing data can be found here: https://www.bristol.ac.uk/staff/researchers/data/accessing-research-data/.

## Software availability statement

No software is required to view the available datasets. The data can be imported into and analysed with any statistical software, including the open source software package R (R Statistical Software (v4.4.1; 2024, https://www.r-project.org/). We used Stata 18 software: StataCorp. 2023. *Stata Statistical Software: Release 18*. College Station, TX: StataCorp LLC. The University of Bristol has a Stata site licence.

## References

[1] Terasaki P Cecka J Gjertson D : High survival rates of kidney transplants from spousal and living unrelated donors. *N Engl J Med.* 1995;333(6):333–6. 10.1056/NEJM199508103330601 7609748

[2] Wyld M Morton R Hayen A : A systematic review and meta-analysis of utility-based Quality of Life in Chronic Kidney Disease treatments. *PLoS Med.* 2012;9(9): e1001307. 10.1371/journal.pmed.1001307 22984353 PMC3439392

[3] NHS Blood and Transplant: Organ donation and transplantation activity Report 2020/21. 2021; [Accessed 24th January 2022]. Reference Source

[4] Cecka J : Living donor transplants. *Clin Transpl.* 1995;363–77. 8794280

[5] Laupacis A Keown P Pus N : A study of the quality of life and cost-utility of renal transplantation. *Kidney Int.* 1996;50(1):235–42. 10.1038/ki.1996.307 8807593

[6] Reese P Boudville N Garg A : Living kidney donation: outcomes, ethics, and uncertainty. *Lancet.* 2015;385(9981):2003–13. 10.1016/S0140-6736(14)62484-3 26090646

[7] Lumsdaine JA Wray A Power MJ : Higher quality of life in living donor kidney transplantation: prospective cohort study. *Transpl Int.* 2005;18(8):975–80. 10.1111/j.1432-2277.2005.00175.x 16008749

[8] Segev DL Muzaale AD Caffo BS : Perioperative mortality and long-term survival following live kidney donation. *JAMA.* 2010;303(10):959–66. 10.1001/jama.2010.237 20215610

[9] Muzaale AD Massie AB Wang M : Risk of End-Stage Renal Disease following live kidney donation. *JAMA.* 2014;311(6):579–86. 10.1001/jama.2013.285141 24519297 PMC4411956

[10] Kortram K Ijzermans J Dor F : Perioperative events and complications in minimally invasive live donor nephrectomy: a systematic review and meta-analysis. *Transplantation.* 2016;100(11):2264–75. 10.1097/TP.0000000000001327 27428715

[11] Najarian J Chavers B McHugh L : 20 years or more of follow-up of living kidney donors. *Lancet.* 1992;340(8823):807–10. 10.1016/0140-6736(92)92683-7 1357243

[12] Johnson E Anderson J Jacobs C : Long-term follow-up of living kidney donors: quality of life after donation. *Transplantation.* 1999;67(5):717–21. 10.1097/00007890-199903150-00013 10096528

[13] Ku J : Health-related quality of life of living kidney donors: review of the short form 36-health questionnaire survey. *Transpl Int.* 2005;18(12):1309–17. 10.1111/j.1432-2277.2005.00231.x 16297049

[14] Garcia MFFM Andrade LGM Carvalho MFC : Living kidney donors--a prospective study of quality of life before and after kidney donation. *Clin Transpl.* 2013;27(1):9–14. 10.1111/j.1399-0012.2012.01687.x 22831164

[15] Axelrod D Schnitzler M Xiao H : An economic assessment of contemporary kidney transplant practice. *Am J Transplant.* 2018;18(5):1168–76. 10.1111/ajt.14702 29451350

[16] Howard K Salkeld G White S : The cost-effectiveness of increasing kidney transplantation and home-based dialysis. *Nephrology (Carlton).* 2009;14(1):123–32. 10.1111/j.1440-1797.2008.01073.x 19207859

[17] NHS Standard Contract for Adult Kidney Transplant Service Schedule 2 - The Services A. Service Specification Number: A07/S/a. Number: A07/S/a,2013. Reference Source

[18] Annema C Op den Dries S van den Berg A : Opinions of dutch liver transplant recipients on anonymity of organ donation and direct contact with the donors family. *Transplantation.* 2015;99(4):879–84. 10.1097/TP.0000000000000394 25211521

[19] International Registry in Organ Donation and Transplantation (IRODaT): Worldwide kidney transplant from living donors 2019 (PMP). [Accessed 8th February 2021]. Reference Source

[20] NHS Blood and Transplant: Organ donation and transplantation activity report 2018/19. [Accessed 4th April 2022]. Reference Source

[21] Eurotransplant Statistics Report Library. Reference Source

[22] Udayaraj U Ben-Shlomo Y Roderick P : Social deprivation, ethnicity, and uptake of living kidney donor transplantation in the United Kingdom. *Transplantation.* 2012;93(6):610–6. 10.1097/TP.0b013e318245593f 22245879

[23] Wu D Robb M Watson C : Barriers to living donor kidney transplantation in the United Kingdom: a national observational study. *Nephrol Dial Transplant.* 2017;32(5):890–900. 10.1093/ndt/gfx036 28379431 PMC5427518

[24] Khalil K Brotherton A Moore S : Interaction between socioeconomic deprivation and ethnicity for likelihood of receiving living-donor kidney transplantation. *BMC Nephrol.* 2022;23(1): 113. 10.1186/s12882-022-02742-6 35305568 PMC8934457

[25] European Renal Best Practice (ERBP) Transplantation guideline development group: Field version ERBP guideline on kidney donor and recipient evaluation and perioperative care.2014; may 2014 [cited 2015 Oct 10].

[26] Frankl FEK Coward RJ Gallagher H : UK renal research strategy.UK Kidney Research Consortium (UKKRC),2016. Reference Source

[27] Bailey PK Ben-Shlomo Y Caskey FJ : Development of an intervention to improve access to living-donor kidney transplantation (the ASK study). *PLoS One.* 2021;16(6): e0253667. 10.1371/journal.pone.0253667 34170946 PMC8232417

[28] Bailey PK Ben-Shlomo Y Tomson CRV : Socioeconomic deprivation and barriers to live-donor kidney transplantation: a qualitative study of deceased-donor kidney transplant recipients. *BMJ Open.* 2016;6(3): e010605. 10.1136/bmjopen-2015-010605 26936910 PMC4785291

[29] Bailey PK Caskey FJ MacNeill S : Mediators of socioeconomic inequity in Living-Donor Kidney Transplantation: results from a UK multicenter case-control study. *Transplant Direct.* 2020;6(4): e540. 10.1097/TXD.0000000000000986 32309626 PMC7145004

[30] Bailey PK Caskey FJ MacNeill S : Beliefs of UK transplant recipients about Living Kidney Donation and Transplantation: findings from a multicentre questionnaire-based case–control study. *J Clin Med.* 2019;9(1):31. 10.3390/jcm9010031 31877750 PMC7019237

[31] Wong K Owen-Smith A Caskey F : Investigating ethnic disparity in Living-Donor Kidney Transplantation in the UK: patient-identified reasons for non-donation among family members. *J Clin Med.* 2020;9(11):3751. 10.3390/jcm9113751 33233422 PMC7700269

[32] Taylor DM Bradley JA Bradley C : Limited health literacy is associated with reduced access to kidney transplantation. *Kidney Int.* 2019;95(5):1244–1252. 10.1016/j.kint.2018.12.021 30952457

[33] Bailey PK Caskey FJ MacNeill S : Investigating strategies to improve AccesS to Kidney transplantation (the ASK trial): a protocol for a feasibility randomised controlled trial with parallel process evaluation. *Pilot Feasibility Stud.* 2023;9(1): 13. 10.1186/s40814-023-01241-1 36670510 PMC9854094

[34] Moore GF Audrey S Barker M : Process evaluation of complex interventions: Medical Research Council guidance. *BMJ.* 2015;350: h1258. 10.1136/bmj.h1258 25791983 PMC4366184

[35] Barnieh L Collister D Manns B : A scoping review for strategies to increase living kidney donation. *Clin J Am Soc Nephrol.* 2017;12(9):1518–1527. 10.2215/CJN.01470217 28818845 PMC5586566

[36] Jakobsen A Albrechtsen D Leivestad T : Renal transplantation--the Norwegian model. *Ann Transplant.* 1996;1(3):32–5. 9869917

[37] Ismail SY Luchtenburg AE Timman R : Home-based family intervention increases knowledge, communication and living donation rates: a randomized controlled trial. *Am J Transplant.* 2014;14(8):1862–9. 10.1111/ajt.12751 24935081

[38] Rodrigue JR Cornell DL Lin JK : Increasing live donor kidney transplantation: a randomized controlled trial of a home-based educational intervention. *Am J Transplant.* 2007;7(2):394–401. 10.1111/j.1600-6143.2006.01623.x 17173659

[39] Garg AX Yohanna S Naylor KL : Effect of a novel multicomponent intervention to improve patient access to kidney transplant and Living Kidney Donation: the EnAKT LKD cluster randomized clinical trial. *JAMA Intern Med.* 2023;183(12):1366–1375. 10.1001/jamainternmed.2023.5802 37922156 PMC10696487

[40] Ministry of Housing, Communities & Local Government: The English Indices of Deprivation 2019 (IoD2019). Sept 26,2019; [Accessed 10 December 2020]. Reference Source

[41] Kayler LK Dolph B Seibert R : Development of the living donation and kidney transplantation information made easy ( *KidneyTIME*) educational animations. *Clin Transpl.* 2020;34(4): e13830. 10.1111/ctr.13830 32072670

[42] Statistics and Clinical Audit: NHS Blood and Transplant Organ Donation and Transplantation Activity Report 2016/17. [Accessed 12 March 2021]. Reference Source

[43] Ismail SY Timmerman L Timman R : A psychometric analysis of the Rotterdam Renal Replacement Knowledge-Test (R3K-T) using item response theory. *Transpl Int.* 2013;26(12):1164–72. 10.1111/tri.12188 24118241

[44] Cohen S : Basic psychometrics for the ISEL-12.2008; [Accessed 12 March 2021]. Reference Source

[45] Merz EL Roesch SC Malcarne VL : Validation of Interpersonal Support Evaluation List-12 (ISEL-12) scores among English- and Spanish-speaking Hispanics/Latinos from the HCHS/SOL Sociocultural Ancillary Study. *Psychol Assess.* 2014;26(2):384–94. 10.1037/a0035248 24320763 PMC4048059

[46] Hibbard JH Mahoney ER Stockard J : Development and testing of a short form of the patient activation measure. *Health Serv Res.* 2005;40(6 Pt 1):1918–30. 10.1111/j.1475-6773.2005.00438.x 16336556 PMC1361231

[47] Hibbard JH Stockard J Mahoney ER : Development of the Patient Activation Measure (PAM): conceptualizing and measuring activation in patients and consumers. *Health Serv Res.* 2004;39(4 Pt 1):1005–26. 10.1111/j.1475-6773.2004.00269.x 15230939 PMC1361049

[48] Morris NS MacLean CD Chew LD : The Single Item Literacy Screener: evaluation of a brief instrument to identify limited reading ability. *BMC Fam Pract.* 2006;7: 21. 10.1186/1471-2296-7-21 16563164 PMC1435902

[49] Brice JH Foster MB Principe S : Single-item or two-item literacy screener to predict the S-TOFHLA among adult hemodialysis patients. *Patient Educ Couns.* 2014;94(1):71–5. 10.1016/j.pec.2013.09.020 24169024

[50] Gourlay WA Stothers L Liu L : Attitudes and predictive factors for live kidney donation in British Columbia. A comparison of recipients and wait-list patients. *Can J Urol.* 2005;12(1):2511–20. 15777488

[51] Oppe M Devlin NJ van Hout B : A program of methodological research to arrive at the new international EQ-5D-5L valuation protocol. *Value Health.* 2014;17(4):445–53. 10.1016/j.jval.2014.04.002 24969006

[52] Braun V Clarke V : Using thematic analysis in psychology. *Qual Res Psychol.* 2006;3(2):77–101. 10.1191/1478088706qp063oa

[53] Harris PA Taylor R Thielke R : Research Electronic Data Capture (REDCap)--a metadata-driven methodology and workflow process for providing translational research informatics support. *J Biomed Inform.* 2009;42(2):377–81. 10.1016/j.jbi.2008.08.010 18929686 PMC2700030

[54] Bailey P : ASK feasibility trial documents.University of Bristol Data Repository,2024. 10.5523/bris.1u5ooi0iqmb5c26zwim8l7e8rm

[55] Bailey P : The ASK feasibility trial: Wellcome Open Research CONSORT checklist.University of Bristol Data Repository,2024. 10.5523/bris.1m3uhbdfdrykh27iij5xck41le

[56] Bailey P : The ASK feasibility trial: CONSORT documents.University of Bristol Data Repository,2024. 10.5523/bris.2iq6jzfkl6e1x2j1qgfbd2kkbb

[57] Bailey P : ASK feasibility trial: QUANT.University of Bristol Data Repository,2024. 10.5523/bris.2b9vlo0wejnsh2nfoa6fka66cx

[58] Bailey P : ASK feasibility trial: QUAL.University of Bristol Data Repository,2024. 10.5523/bris.1qm9yblprxuj2qh3o0a2yylgg

