# Supplementary material for: The ASK trial: a randomised controlled feasibility trial and process evaluation of a complex multicomponent intervention to improve AccesS to living-donor Kidney transplantation
Source: Wellcome Open Res. 2024 Oct 29;9:628. [Version 1] doi: 10.12688/wellcomeopenres.22631.1 (PMC12457887; doi:10.12688/wellcomeopenres.22631.1)
Supplement: Peer review history

# Reviewer report: Reviewer response for version 1

**DOI:** 10.21956/wellcomeopenres.24932.r132888

**Published:** 2025-09-25

**Authors:** Kristin Veighey

**Affiliations:** 1 University of Southampton, Southampton, England, UK

This is a well written paper and all the sections are thoroughly considered. Reading the protocol paper and intervention development paper was important to understand the project in full, as there were aspects not explained in this paper which were referenced to the protocol or the previous intervention development work. 

 Minor comments:

 1. The abstract does not explain the purpose of the work, this is however clearly articulated in the plain english summary. It would be useful to include a line describing the intention of the multicomponent intervention to the abstract. 

 2. Healthcare systems in Norway and the Netherlands are referenced in the context of interventions that are in place, but there is no explanation of how systematically these countries might differ from the UK in terms of healthcare and transplant delivery models, and how that might impact the choice of intervention - and downstream implementation in the UK. Geography certainly impacts in Norway which has a single transplant centre, making letters a practical option. What was the impact of geography on recruitment (if any)?

 3. There is no discussion up front around ethical considerations - the results discuss these issues such as whether people felt pressure to donate, but there is no up front acknowledgement that doctors writing to people to encourage them to donate could be seen as inappropriate. It is clear the project sought to explore this, but perhaps this should be stated in the introductory sections. I note this was explored in the development of the intervention through qualitative work, but perhaps a brief mention here would be useful. 

 4. All aspects of the intervention were retained yet the most acceptable interventions by far were 1 and 2. Yet the conclusion states the overall intervention was acceptable. Should 3 have been retained? In the intervention development paper the 3 aspects were proposed and refined, but perhaps the overall intervention could be further streamlined?

 3. There are no details on the cost of the intervention and any consideration to the labour intensive parts 1 and 3 in terms of workforce. Although undoubtedly cost effective if successful in terms of one patient receiving a transplant vs dialysis, the cost of each element would be useful information to determine whether this could be practical to implement. I presume a full HE analysis forms part of the subsequent study.  

 5. Diversity is documented in terms of ethnicity, yet the introduction states that deprivation drives ethnic inequity, and this is explored further in the initial intervention development paper. It would have been useful to understand whether those living in areas of deprivation were included.

Is the work clearly and accurately presented and does it cite the current literature?

Yes

If applicable, is the statistical analysis and its interpretation appropriate?

Yes

Are all the source data underlying the results available to ensure full reproducibility?

Yes

Is the study design appropriate and is the work technically sound?

Yes

Are the conclusions drawn adequately supported by the results?

Yes

Are sufficient details of methods and analysis provided to allow replication by others?

Yes

Reviewer Expertise:

1. Risk stratification of CKD in primary care, 2. Prehabilitation and preconditioning for kidney transplant recipients, 3. Health inequalities in primary care and impact on CKD care

I confirm that I have read this submission and believe that I have an appropriate level of expertise to confirm that it is of an acceptable scientific standard.

# Reviewer report: Reviewer response for version 1

**DOI:** 10.21956/wellcomeopenres.24932.r132893

**Published:** 2025-09-23

**Authors:** Adnan Sharif

**Affiliations:** 1 University of Birmingham, Birmingham, UK

This work summarises a feasibility study that explores a home based living donor kidney education intervention versus standard of care. It shows the feasibility of increased engagement and approaches for further information (28% versus 10%) in a representative cohort. 

 There is definite merit in extending this feasibility study to UK units to try and boost living donor kidney procedures, as the authors correctly highlight UK LDKT rates are sub-optimal in comparison to other countries.

 One issue is the workforce required to deliver the intervention. The home based family education and engagement requires a trained professional and trained living kidney donor. In the feasibility study this was a living kidney donor specialist nurse but this may not be possible at each renal transplant unit. The future work section mentions identifying and training a pool of specialist and it is not clear if this includes trained CKD nurses as many units do have skilled CKD specialist nurses who do home education about renal replacement therapy options.

 It will be interesting to see how this study behaves in other centres with greater diversity and it is encouraging that ethnic minority recruitment in the feasibility study was representative.

Is the work clearly and accurately presented and does it cite the current literature?

Yes

If applicable, is the statistical analysis and its interpretation appropriate?

Yes

Are all the source data underlying the results available to ensure full reproducibility?

Yes

Is the study design appropriate and is the work technically sound?

Yes

Are the conclusions drawn adequately supported by the results?

Yes

Are sufficient details of methods and analysis provided to allow replication by others?

Yes

Reviewer Expertise:

Kidney transplantation

I confirm that I have read this submission and believe that I have an appropriate level of expertise to confirm that it is of an acceptable scientific standard.

# Reviewer report: Reviewer response for version 1

**DOI:** 10.21956/wellcomeopenres.24932.r108982

**Published:** 2024-11-26

**Authors:** Helen Noble

**Affiliations:** 1 Queen's University Belfast, Belfast, UK

This is a very well written paper on a randomised controlled feasibility trial and process evaluation of a complex multicomponent intervention to improve AccesS to living-donor Kidney transplantation. The feasibility trial follows on from a mixed-methods intervention development study where the authors developed a multicomponent intervention to address previously identified barriers to living-donor kidney transplantation. The trial comprises: 1.  A meeting between a home educator and the transplant candidate with a discussion about living-donor kidney transplantation and living kidney donation, and potential donors. 2.  A standardized letter and information sheet from a healthcare professional to a candidate’s potential donors. 3.  A home-based education and family engagement session with the transplant candidates and their family/friends, undertaken by two home educators: a living kidney donor and a nurse specialist. The feasibility trial was designed to gather information on the acceptability, logistics of delivery, and risk of harm of a multicomponent intervention designed to improve equitable access to LDKTs. It aimed to determine if it would be possible to conduct a full-scale effectiveness trial, and to determine the parameters needed to design such a trial. A parallel process evaluation investigated the acceptability, implementation and mechanisms of impact of the intervention.

 As stated by the authors the feasibility trial demonstrated that the multicomponent intervention and trial processes are i) acceptable to patients, their families and healthcare professionals, and ii) deliverable in transplanting and non-transplanting referral NHS hospitals in the UK. The study demonstrated that a larger effectiveness trial is feasible and is is excellent news that a decision has been made to progress to an effectiveness RCT.

 I have added some comments and suggested changes to the attachment. I have suggested that the process evaluation results are presented more clearly in terms of themes and reference back to the table included.

 Please view the additional review comments in the file found here

Is the work clearly and accurately presented and does it cite the current literature?

Yes

If applicable, is the statistical analysis and its interpretation appropriate?

I cannot comment. A qualified statistician is required.

Are all the source data underlying the results available to ensure full reproducibility?

Yes

Is the study design appropriate and is the work technically sound?

Yes

Are the conclusions drawn adequately supported by the results?

Yes

Are sufficient details of methods and analysis provided to allow replication by others?

Yes

Reviewer Expertise:

Professor of Nursing. Health and well-being of people with kidney disease and their carers.

I confirm that I have read this submission and believe that I have an appropriate level of expertise to confirm that it is of an acceptable scientific standard.

# Response

**Published:** 2024-11-29

**Authors:** Pippa Bailey

**Affiliations:** Bristol Medical School: Population Health Sciences, University of Bristol, Bristol, England, UK

Thank you for such a comprehensive and valuable review. We will work through your suggestions and address your queries in order to improve the report. Thank you for the time you will have spent on this. Best wishes, Pippa
